# Lumbar clear cell meningioma mimicking schwannoma 7 years after resection of the same type of intracranial tumor: a case report

**DOI:** 10.1186/s13256-024-04411-8

**Published:** 2024-02-06

**Authors:** Tomoyuki Horikawa, Satoshi Nozawa, Natsuko Suzui, Kazunari Yamada, Chizuo Iwai, Haruhiko Akiyama

**Affiliations:** 1 Department of Orthopaedic Surgery, Gifu University School of Medicine, 1-1 Yanagido, Gifu, Gifu 501-1194 Japan; 2 Department of Orthopaedic Surgery, Mino Municipal Hospital, Mino, Japan; 3 Department of Pathology, Gifu University Hospital, Gifu, Japan

**Keywords:** Clear cell meningioma, Mimicking schwannoma, Intracranial, Intraspinal, SMARCE1

## Abstract

**Background:**

Meningioma is the second most common intradural extramedullary tumor, following schwannoma. Meningioma is primarily categorized as benign World Health Organization grade 1, but clear cell meningioma is grade 2 of the intermediate malignant category. Clear cell meningiomas are rare, accounting for less than 1% of all meningioma tumors. There is no previous report of multiple intraspinal clear cell meningiomas without dural attachment.

**Case presentation:**

A 27-year-old Asian male patient presented with lower right extremity pain, and had undergone tumor resection for intracranial clear cell meningioma 7 years previously, with re-resection and radiotherapy for local tumor recurrence at our hospital’s department of neurosurgery being carried out 4 years previously. No recurrence was observed since then. Preoperative lumbar magnetic resonance imaging showed two tumors at the L1 and L4 levels, both mimicking schwannoma with well-defined margins, no dural tail sign and homogeneous internal contrast. Intraoperative findings on tumor resection showed two tumors contiguous with the right L2 and L5 roots, which were not attached to the dura mater, similar to a schwannoma. After gross total resection, the postoperative pathology revealed no nuclear SMARCE1 antibody staining. The patient was diagnosed with clear cell meningioma. The patient’s postoperative course went well, with no symptoms of nerve dropout and no recurrence 2 years after surgery. In this case, both lumbar lesions were well demarcated and spherical in shape, occurring with single roots. Tumor characteristics suggested a primary rather than a metastatic lesion. Clear cell meningioma is characterized by a *SMARCE1* mutation and is different from other types of meningiomas.

**Conclusion:**

To the best of our knowledge, this is the first report of multiple intraspinal clear cell meningiomas without dural attachment at the lumbar spine after resection of intracranial clear cell meningioma. We speculate that the two tumors were de novo lesions on the basis of the features of the tumors, although they were detected 7 years after the resection of intracranial clear cell meningioma.

## Background

Clear cell meningioma (CCM) is an unusual histologic strain of meningioma, accounting for only 0.2% of all meningiomas [1]. The pathologic feature of this tumor is the presence of clear cytoplasm in the tumor cells due to glycogen accumulation. Pathologic identification remains essential for the diagnosis of CCM, though a novel mutation in *SMARCE1*, encoding a subunit of the SWI/SNF chromatin remodeling complex, is linked to the development of CCM [2–5]. CCMs occur most commonly in the cerebellopontine angle [6]. Intraspinal CCMs are very rare, and less than 100 cases have been reported in the English literature [7]. CCMs without dural adhesions are even rarer, with only 20 cases reported [8].

We report the first case of multiple nondura-based clear cell meningiomas without SMARCE1 expression at the lumbar spine 7 years after resection of intracranial CCM. It is speculated that the two intraspinal tumors were de novo lesions due to loss-of-function mutations in *SMARCE1*, resulting in inherited disorder of multiple spinal meningiomas [3]. If CCM is detected in the intracranial or intraspinal lesion, close observation is required to determine whether other CCMs occur in the lesion.

## Case presentation

**Fig. 1:**
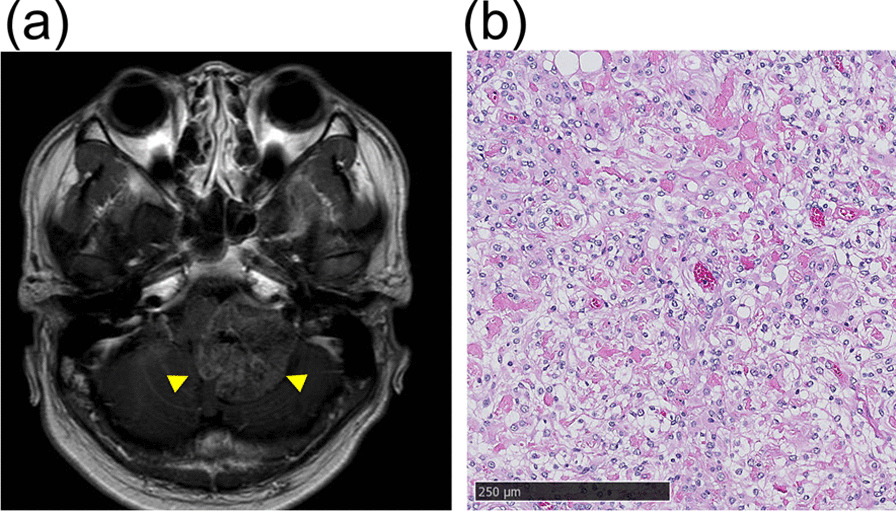
Before the initial brain surgery, head gadolinium-enhanced T1-weighted magnetic resonance imaging (MRI) showing a tumor (arrow heads) located in the left cerebellopontine angle (**a**). Histological evaluation of the initial intracranial tumor revealed a sheeting architecture containing round to polygonal cells with clear cytoplasm and many small clumps of collagen fibers (**b**). The tumor was diagnosed as clear cell meningioma

A 27-year-old Asian male patient was referred to our hospital after experiencing pain in his right leg over a period of 3 months. He had no family and psychosocial history. His medical history is as follows. At the age of 20 years, he first visited the neurosurgeon’s office for chronic headache. No clinical findings were observed in his extremities. Head gadolinium-enhanced T1-weighted magnetic resonance imaging (MRI) showed a tumor located in the left cerebellopontine angle (Fig. 1a). Surgery was performed for resection followed by histological evaluation of the tumor, which revealed a sheeting architecture containing round to polygonal cells with clear cytoplasm and many small clumps of collagen fiber. The tumor was diagnosed as CCM (Fig. 1b). When the patient was 23 years old, local recurrence of the tumor was detected adjacent to left foramen of Luschka. Re-resection was performed followed by radiotherapy 38.0 Gy in 10 fractions. No local recurrence and no symptoms were observed for the following 4 years.

**Fig. 2:**
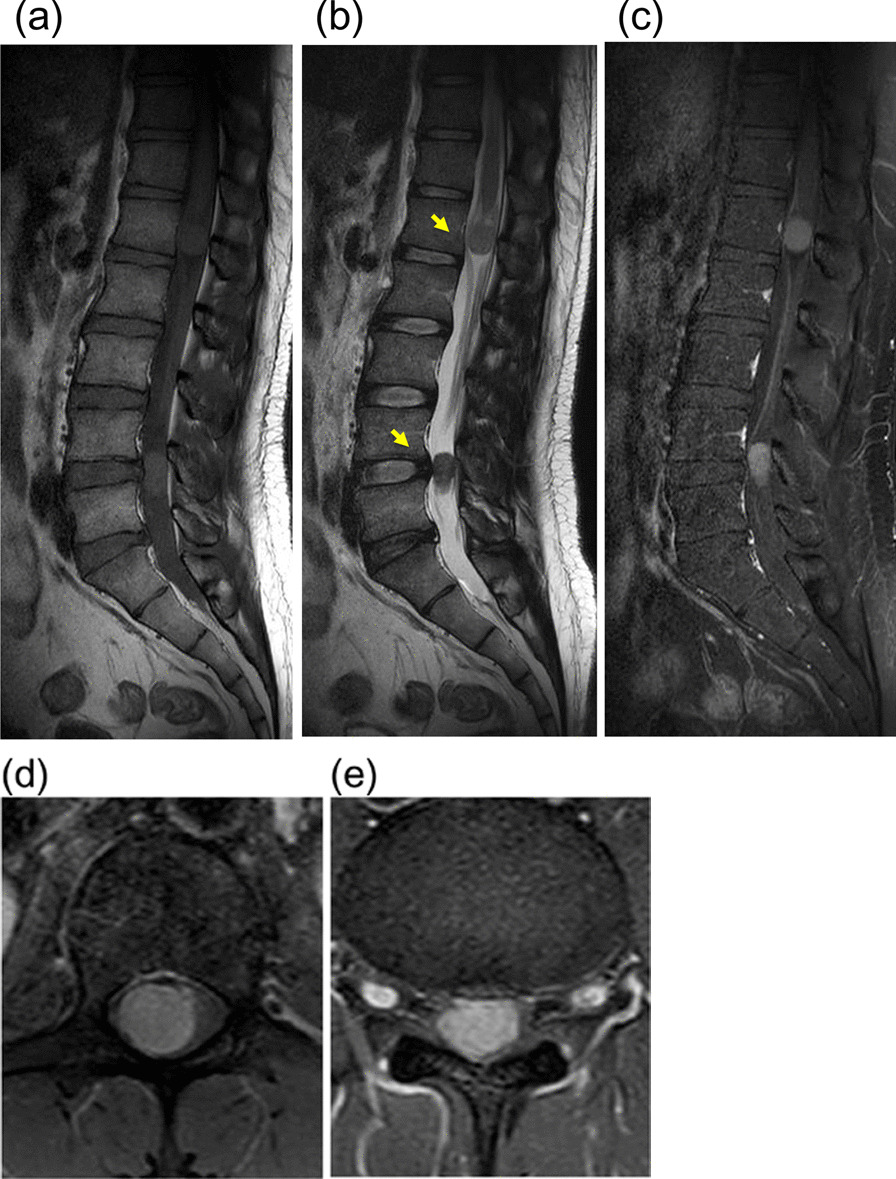
Lumbar MRI showing two intradural masses (arrows) without “dural tail signs” at the L1 and L4/L5 levels on the T1-weighted image (**a**) and T2-weighted image (**b**). Gadolinium-enhanced T1-weighted MRI images showed the masses with homogeneous enhancement on the sagittal image (**c**) and the axial images at the L1 (**d**) and at L4/5 (**e**) levels

**Fig. 3:**
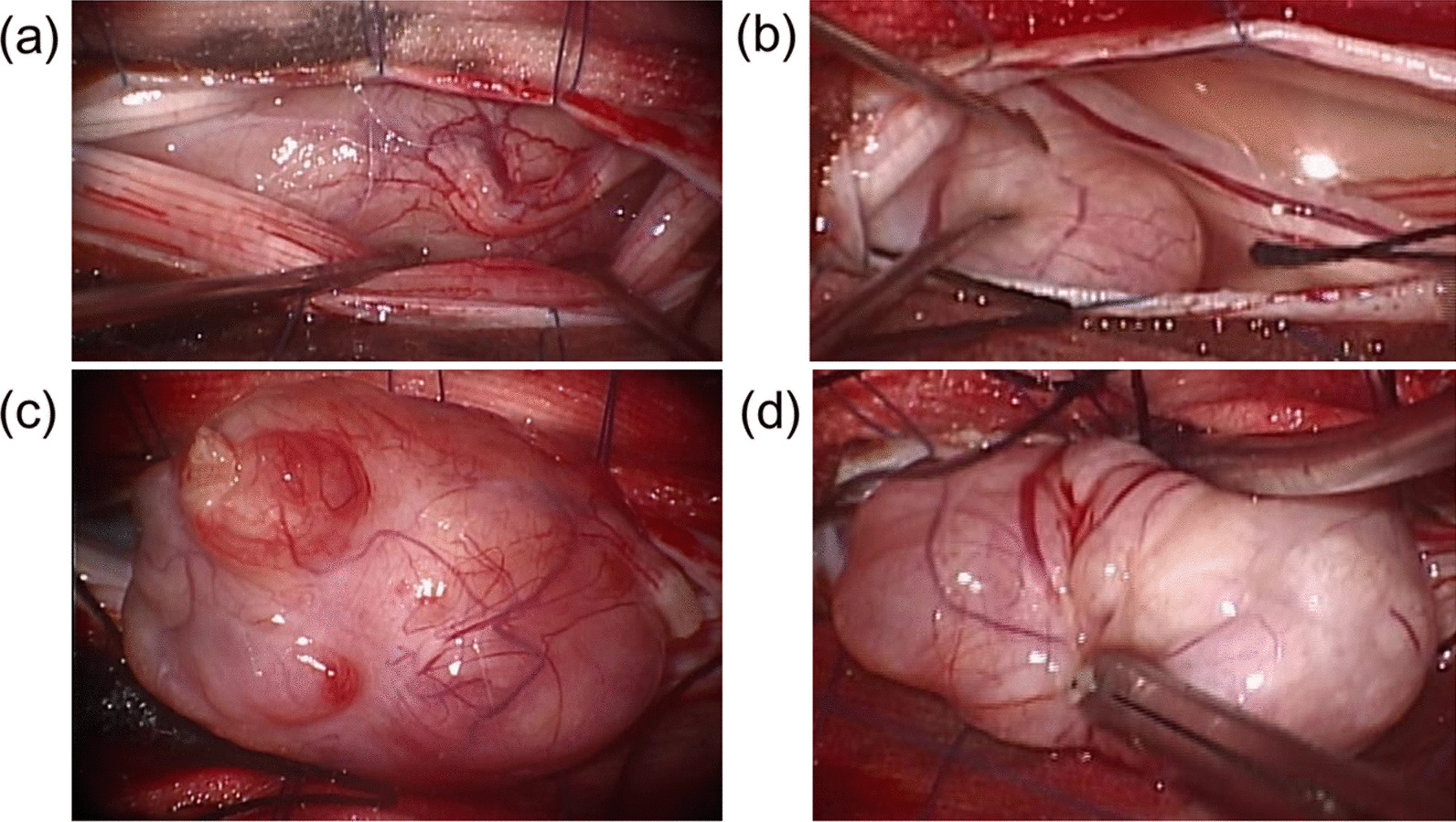
Tumors observed growing separately in the right L1 (**a**, **c**) and L5 (**b**, **d**) roots after T12-L1 and L4-5 laminectomy, and no dural attachment detected, mimicking schwannoma

At the age of 27 years, the patient complained of pain in his right lower extremity and required the assistance of a cane to walk. No muscle weakness, bowel dysfunction, or bladder dysfunction were detected. The Japanese Orthopaedic Association score (maximum score of 29 points) was 20 before the surgery. No intracranial tumor was detected. A lumbar MRI showed two intradural masses without “dural tail signs” at the L1 and L4/L5 levels (Fig. 2a, b). Gadolinium-enhanced T1-weighted MRI images showed homogeneous enhancement of the masses (Fig. 2c–e). Surgery was performed 3 months after the patient’s first examination in our department. After T12-L1 and L4-5 laminectomy, tumor margins underneath the dura mater were identified using ultrasound, and the dura was split. Under the arachnoid membrane, the tumors were growing separately in the right L1 (Fig. 3a, c) and L5 (Fig. 3b, d) roots and no dural attachment was detected, mimicking schwannoma. Complete total resection of the tumors was performed after severing the afferent and efferent nerves. The tumor was covered with a capsule, and no adhesion to surrounding tissue was observed.

**Fig. 4:**
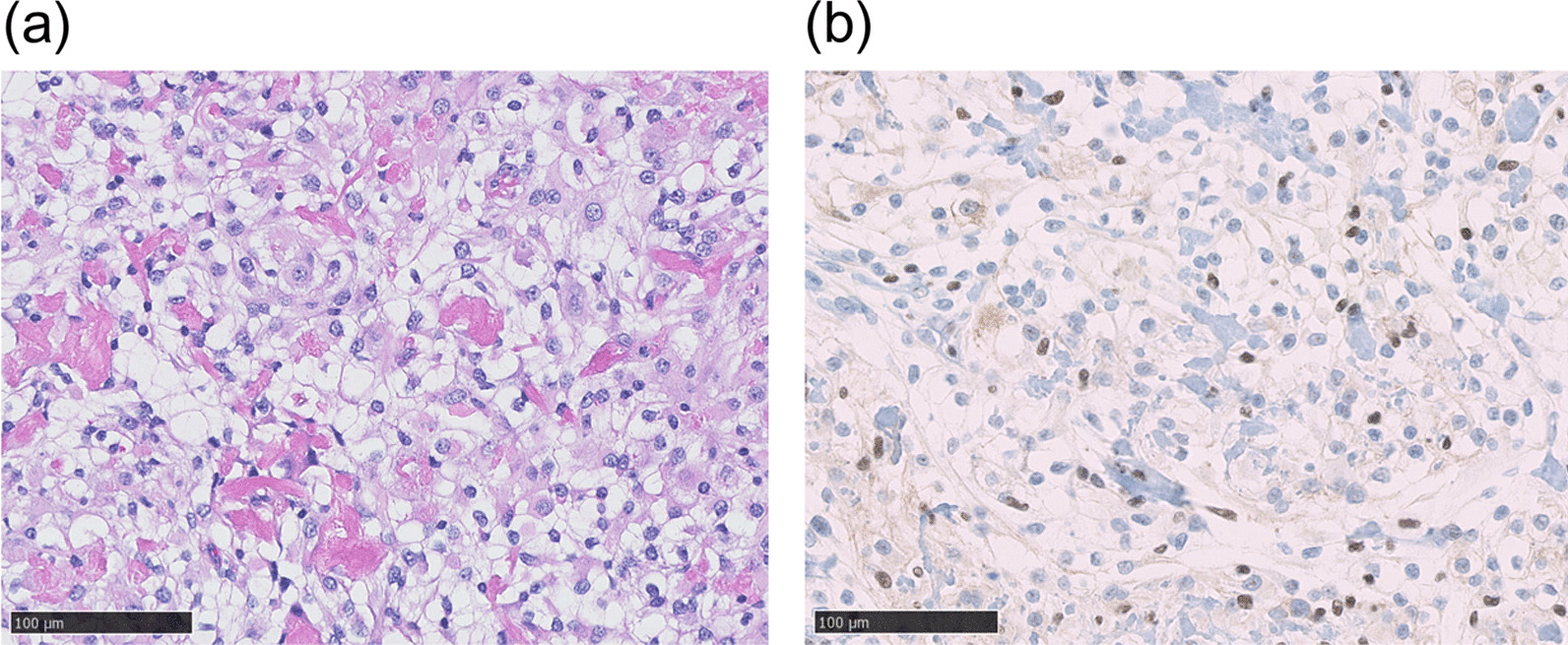
Tumor exhibiting sheeting architecture containing round to polygonal cells with clear cytoplasm and many small clumps of collagen fibers histologically. Whorl formation and psammoma bodies were poorly formed (H and E stain) (**a**). Immunohistochemistry revealed the loss of nuclear SMARCE1 expression in the tumor cells (SMARCE1) (**b**)

Histologically, the tumor showed sheeting architecture containing round to polygonal cells with clear cytoplasm and many small clumps of collagen fiber. Whorl formation and psammoma bodies were poorly formed (Fig. 4a). A few peripheral nerves were involved. Immunohistochemistry revealed nuclear loss of SMARCE1 expression in the tumor cells (Fig. 4b). The pathology of the intracranial CCM that occurred 7 years prior and this intraspinal CCM were similar (Figs. 1c, 4a).

The postoperative course was uneventful. The pain and numbness of the right lower extremity were completely alleviated. No recurrence was observed 2 years after surgery.

## Discussion

CCM was first described by Manivel in 1990 [9]. Although most meningiomas are grade I, CCMs are categorized as grade II because of their aggressive behaviour [10, 11]. CCMs account for 0.2% of all meningioma cases [1]. CCM most commonly occurs in the cerebellopontine angle [6]. Intraspinal CCMs are very rare, and less than 100 spinal CCMs have been reported in English literature [7]. In our case, two schwannoma-like intraspinal CCMs were detected 7 years after intracranial CCM.

The intraspinal CCM in our case was probably not a metastasis for the following reasons. First, meningioma metastases are rare, accounting for only 0.1% of all cases. The most common sites of metastases are the lungs, liver, lymph nodes, and bones; intraspinal metastases are rare [12]. Only one report showed two CCM intracranial and intraspinal lesions and both of the lesions were adherent to the dura mater [13]. Adhesion may be associated with metastases. Second, in our case, the tumors were well defined and rounded, and each tumor was located in a single root only. Further, spinal cord metastases of malignant meningioma are usually irregularly shaped with indistinct borders [14, 15]. Maamri *et al*. reported that some spinal CCMs do not show dural attachment [8]; non-adherent CCMs are very rare, as only 20 cases have been reported in the English literature. In those cases, no metastases were reported. On the basis of these findings, the intraspinal lesions in our case may be multiple de novo tumors rather than metastases.

Histologically, our case study tumor showed sheeting architecture containing round to polygonal cells with clear cytoplasm and many small clumps of collagen fibers. Whorl formation and psammoma bodies were poorly formed and consistent with previous reports of spinal CCM [7, 8]. However, it is noteworthy that immunohistochemically, nuclear loss of the SMARCE1 expression in the tumor cells was confirmed in our case. Recent research showed that a loss of SMARCE1 expression is a specific diagnostic marker of CCM [2]. The tumorigenesis of meningiomas, derived from arachnoid cells, depends on various germline and somatic gene mutations. The most common mutations are germline NF2 mutations, which are responsible for neurofibromatosis type 2 (NF2) [16]. Two genes (*SMARCB1* and *SMARCE1*) encoding two proteins of the SWI/SNF complex (BAF47 and SMARCE1) have also been proven to be the primary cause of hereditary meningiomatosis [2, 16]. In CCM, mutations in the SMARCE1 gene occurred in 33 out of 34 cases, and unlike other meningiomas, CCM tumors are derived from progenitor cells [17]. Sievers *et al*. speculated that these tumors may arise from a different precursor cell population than other meningioma subtypes [17]. On the basis of these facts, we cannot rule out the possibility that genetic factors may have contributed to the multiple intraspinal lesions in this case. Briefly, SWI/SNF subunit mutation due to loss of SMARCE1 might cause tumorigenesis in neural stem cells. Further work is required to elucidate the mechanism of SMARCE1-associated meningioma development.

Tao *et al*. reported that total resection of spinal cord CCM promoted a lower relapse rate. They suggested that radiation therapy need not be performed immediately after the first operation [18]. We also did not use radiotherapy. However, the 1-, 5-, and 10-year recurrence-free survival rates after resection of spinal CCMs are reported to be 87%, 71%, and 47%, respectively [7]. Thus, these tumors should be carefully monitored.

## Conclusion

We experienced a very rare case of multiple intraspinal CCMs 7 years after the initial resection of an intracranial CCM. The tumors were nondura-based meningiomas of the lumbar spine. Genetic factors, such as loss-of-function mutations in *SMARCE1*, may influence multiple occurrences of intracranial and intraspinal lesions. If CCM is detected in an intracranial or intraspinal lesion, close observation is recommended, with the possibility of other CCMs occurring in the lesion.

## Data Availability

Data are available on reasonable request to corresponding author.

## Abbreviations

CCM: Clear cell meningioma
MRI: Magnetic resonance imaging
